# COGNITIVE EFFECTS OF PM2.5 EXPOSURE FROM MID-LIFE TO EARLY OLD AGE

**DOI:** 10.1093/geroni/igac059.2297

**Published:** 2022-12-20

**Authors:** Carol Franz, Xin Tu, Yongmei Qin, Jeremy Elman, Christine Fennema-Notestine, Jaden DeAnda, Tsung-Chin Wu, William Kremen

**Affiliations:** University of California San Diego, La Jolla, California, United States; University of California San Diego, La Jolla, California, United States; University of California San Diego, La Jolla, California, United States; University of California San Diego, La Jolla, California, United States; University of California San Diego, San Diego, California, United States; San Diego State University, La Jolla, California, United States; University of California, La Jolla, California, United States; University of California San Diego, La Jolla, California, United States

## Abstract

Air pollution exposure is a notable public health hazard with adverse effects on multiple health outcomes as well as with increased risk of developing cognitive impairment, Alzheimer’s disease, and related dementias. Few studies examine associations between air pollution exposure in midlife or the transition from midlife to old age. We examined associations between exposure in midlife and cognitive functioning in early old age in ~800 men from the Vietnam Era Twin Study of Aging. Measures included PM2.5 and NO2 exposure in the three years prior to the time 1 (mean age 56; range 51-61) assessment, and cognitive performance in 5 domains at time 1 and time 2 (mean age 68; range 65-72). Analyses adjusted for multiple health and lifestyle covariates. Cognitive performance in all domains was worse at age 68 than at age 56. There was a main effect of midlife PM2.5 on verbal fluency; greater PM2.5 exposure was associated with worse fluency. This association was at a trend level for NO2. In addition, we found significant PM2.5-by-APOE genotype interactions. Increased exposure to PM2.5 in midlife was related to lower executive function and working memory performance in APOE-ε4 carriers, but not non-carriers. Both early executive deficits and APOE-ε4 status have been associated with increased risk for progression to mild cognitive impairment and Alzheimer’s disease. The present results indicate that midlife PM2.5 exposure in men is an additional factor contributing to poorer frontal-executive function, and that APOEε4 carriers are more susceptible to the deleterious effects of PM2.5.

